# The Novel Effect and Potential Mechanism of Lactoferrin on Organ Fibrosis Prevention

**DOI:** 10.3390/nu17010197

**Published:** 2025-01-06

**Authors:** Yonghui Yu, Juan Fang, Yingying Li, Xueli Wang, Jingjie Zhang, Jing Wang, Baoguo Sun

**Affiliations:** Key Laboratory of Geriatric Nutrition and Health, Ministry of Education, China-Canada Joint Lab of Food Nutrition and Health, Key Laboratory of Special Food Supervision Technology for State Market Regulation, Beijing Technology and Business University, Beijing 100048, China; yonghuiwh@126.com (Y.Y.); fangjuan991129@163.com (J.F.); liyingying_siren@163.com (Y.L.); wangxueli521856@163.com (X.W.); sunbg@btbu.edu.cn (B.S.)

**Keywords:** lactoferrin, organ fibrosis, inflammation, oxidative stress, ECM, microbiota, miRNA

## Abstract

Organ fibrosis is gradually becoming a human health and safety problem, and various organs of the body are likely to develop fibrosis. The ultimate pathological feature of numerous chronic diseases is fibrosis, and few interventions are currently available to specifically target the pathogenesis of fibrosis. The medical detection of organ fibrosis has gradually matured. However, there is currently no effective treatment method for these diseases. Therefore, we need to strive for developing effective and reliable drugs or substances to treat and prevent fibrotic diseases. Lactoferrin (LF) is a multifunctional glycoprotein with many pathological and physiologically active effects, such as antioxidant, anti-inflammatory and antimicrobial effects, and it protects against pathological and physiological conditions in various disease models. This review summarizes the effects and underlying mechanisms of LF in preventing organ fibrosis. As a naturally active substance, LF can be used as a promising and effective drug for the prevention and remission of fibrotic diseases.

## 1. Introduction

LF, which is rich in cow colostrum and human milk, is an 80 kDa iron-binding glycoprotein in the transferrin family that exists primarily in milk and other external secretions, in addition to the secondary granules of neutrophils [1,2]. Structurally, LF is a globular glycoprotein composed of two homologous domains, called the N- and C-terminal lobes. The glycosylation of these lobes is uneven (the C-lobe usually contains more N-linked glycosylation sites) [3]. Each lobe can simultaneously combine a single iron ion and a bicarbonate anion. LF and its lobes have extensive antibacterial and antiviral activities. Generally, the N-lobe has the most antibacterial activity [4]. It also has antioxidant, antitumour, anti-inflammatory, and immune-boosting benefits and is commonly used in nutritional health products, infant formula milk powder, auxiliary treatments for metabolic diseases, and food additives [5]. 

Fibrosis is a pathological process whose features are caused by the excessive accumulation of extracellular matrix (ECM), such as collagen, fibronectin, and hyaluronic acid, which occurs during chronic inflammation [6]. Fibrosis is the result of a dysregulated tissue repair response after multiple tissue injuries and is more evident in chronic inflammatory diseases [7]. This dynamic response is composed of three main processes: the primary inflammatory response, the regulation of effector cell activation, and the excessive production of ECM proteins [8]. Many organs can become fibrotic, including the liver, kidneys, lungs, heart, blood vessels, eyes, pancreas and skin [8]. The liver and lung are two organs that are vulnerable to fibrosis. Chronic liver disease (CLD) may further develop into liver fibrosis, cirrhosis, and even hepatocellular carcinoma. CLD and cirrhosis cause approximately 44,000 deaths every year in the United States and approximately 2 million deaths globally [9,10]. Idiopathic pulmonary fibrosis (IPF), which often occurs in middle-aged and elderly people, is the most widespread interstitial lung disease (ILD) and accounts for 17% to 86% of ILD cases. Approximately 3 million patients around the world suffer from IPF [11]. The continued progression of organ fibrosis can cause destruction of the organ structure, a decline in organ function, and even organ failure, posing a serious danger to human survival and health.

To date, two main methods have been used for alleviating organ fibrosis: pharmacologic treatment and nonpharmacologic treatment. Notably, some natural products, such as alkaloids [12], polysaccharides [13], flavonoids [14], peptides [15], terpenes and polyphenols [16] have been found to have antifibrotic activity. Studies also indicate that LF plays essential roles in alleviating liver and cardiac fibrosis [17,18], indicating that dietary nutrition intervention might be a novel strategy for preventing organ fibrosis. However, complex factors and signalling pathways are connected to the occurrence and development of organ fibrosis, and the molecular mechanisms by which LF prevents organ fibrosis still need to be further revealed. This review focuses on the potential of LF for preventing organ fibrosis, summarizes the critical effects of LF on reducing inflammation and antioxidative activity and regulating the synthesis and degradation of the ECM, and proposes new strategies for preventing LF fibrosis, including microbiome regulation and alterations in the profiles of noncoding RNA (such as miRNA and circRNA) profiles.

## 2. Potency of Lactoferrin for Preventing Organ Fibrosis

**Figure 1:**
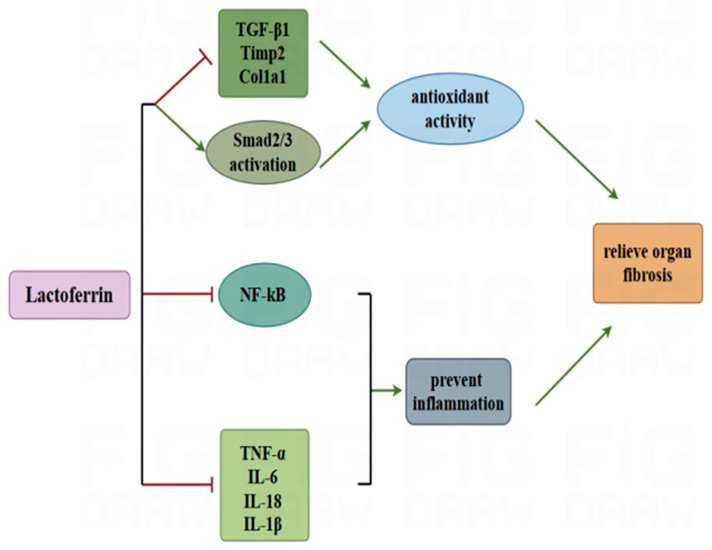
LF prevents organ fibrosis partially by inhibiting inflammation and oxidation. LF can downregulate the expression of inflammatory factors (TNF-α, IL-6, IL-18, and IL-1β) and the phosphorylated nuclear factor NF-κB protein, preventing inflammation; at the same time, it can also suppress the activation of smad2/3 and significantly enhance antioxidant activity by downregulating the expression of cytokine mRNAs related to fibrosis (*TGF-β1*, *Timp2*, and *Col1a1*). Ultimately, it can prevent and alleviate organ fibrosis. In the figure, the green arrow represents promotion, and the red line represents inhibition.

Many common chronic diseases can cause organ fibrosis, including diabetes [19], hypertension [20], viral hepatitis [21], heart failure [22], cardiomyopathy [23], idiopathic lung disease [24], scleroderma [25] and cancer [26]. Fibrosis accounts for nearly 45% of all deaths in developed countries [27]. At present, there is a gradual increase in the awareness of active dietary ingredients in the prevention and intervention of organ fibrosis. LF, a well-known functional protein, possesses antioxidant [28], anti-inflammatory [28], antibacterial [29], antiviral [30] and other functional activities [7] and has been widely studied for disease prevention. Forty-eight male *Cx32ΔTg rats* (7 weeks old) were divided into a control group receiving normal drinking water and an experimental group receiving drinking water supplemented with LF (100 or 500 mg/kg) for 17 weeks. The levels of pro-inflammatory cytokines (TNF-α, IL-6, IL-18, and IL-1β) and the expression of fibrosis-related genes (*TGF-β1*, *Timp2*, and *Col1a1*) in the experimental group of rats with non-alcoholic steatohepatitis were significantly suppressed through inactivation of the NF-κB signalling pathway. (Figure 1) [17]. In the *NZB/WF1 mouse* model of systemic lupus erythematosus (SLE), mice in the low-LF diet group were able to significantly increase antioxidant activity and inhibit hepatic fibrosis by decreasing the expression of fibrotic cytokines and Smad2/3 activation [31]. 

Silymarin (SM) is considered an effective antioxidant and liver protection drug [32]. In a thioacetamide-induced liver fibrosis model in rats, oral administration of LF (200 mg/kg/day) and SM (50 mg/kg/day) significantly reduced collagen fibre deposition, decreased the expression of TGF-β1, and improved the grade of cirrhosis in both experimental groups [33]. 

*Burkholderia cenocepacia* is a gram-negative bacterium involved as a respiratory pathogen in cystic fibrosis patients [34]. Reports have indicated that bovine LF can link to the cable pili of *Burkholderia cenocepacia* and inhibit the linking of bacteria to mucins [29]. LF can promote the protection of cystic fibrosis patients against onion spore bacillus infection by blocking bacterial colonization (Figure 1) [29]. The association between COVID-19 infection and acute respiratory distress syndrome has been extensively reported [35]. It can also lead to pulmonary fibrosis (known as post-COVID-19 pulmonary fibrosis), and the progressive consequences of pulmonary fibrosis usually lead to decreased lung function, even resulting in death [36,37]. The potential mechanisms of the antiviral activity of bovine or human LF include direct attachment to viral proteins, blocking virus entry into receptors or inhibiting viral enzyme activity [38,39]. 

Currently, methods for enhancing LF stability have also been studied, and green-synthesized zinc nanoparticles with LF (LF-Zn-NPs) have been established [40]. The results indicated that LF-Zn-NPs could act against SARS-CoV-2 by directly neutralizing or preventing viral replication after infection (Figure 1) [40]. In general, LF has numerous physiological activities, including anti-inflammatory, antioxidant, antibacterial, and antiviral properties, indicating its great potential in preventing organ fibrosis.

## 3. Classical Mechanisms of Lactoferrin Preventing Organ Fibrosis

### 3.1. Suppressing the Inflammatory Response

**Figure 2:**
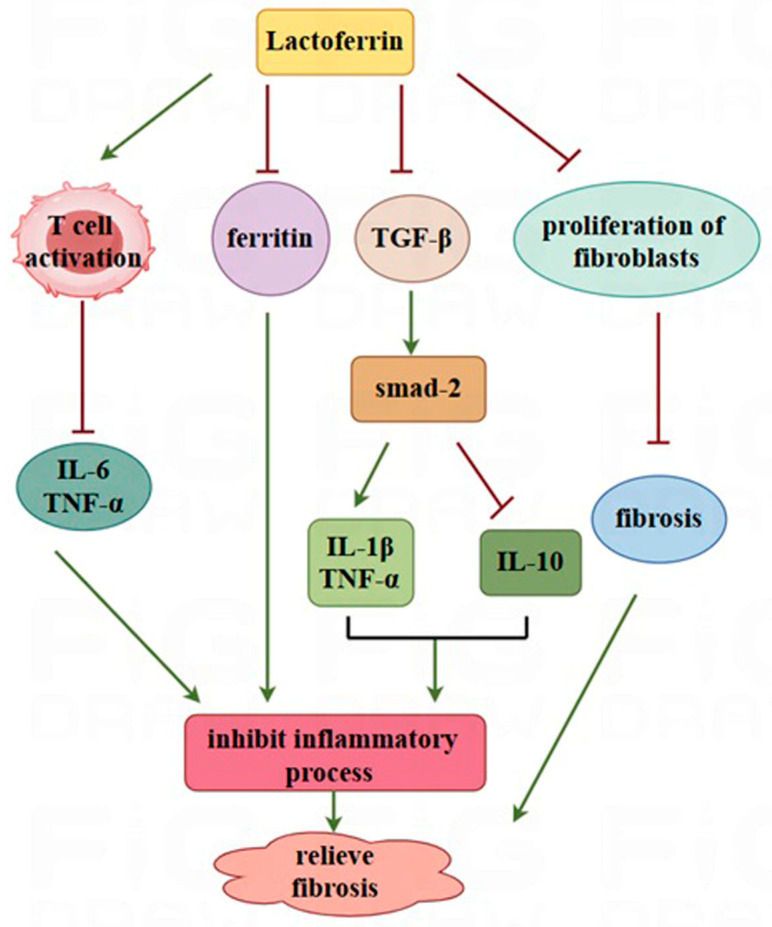
Potential effects of inflammatory factor-mediated LF prevention of organ fibrosis. LF can induce T-cell activation, thereby inhibiting the expression of IL-6 and TNF-α and downregulating ferritin; it can also upregulate the expression of TNF-α and IL-1β and downregulate the expression of IL-10 by activating the TGF-β1/smad-2 pathway. In addition, it can directly inhibit the proliferation of myofibroblasts, subsequently preventing fibrosis. In the figure, the green arrow represents promotion, and the red line represents inhibition.

Excessive inflammatory reactions are among the main factors triggering organ fibrosis. Inflammation is a double-edged sword, and a moderate inflammatory reaction is critical for tissue repair and regeneration; however, an excessive inflammatory response is involved in the occurrence of fibrosis. In damaged organs, chemotactic stimulation triggers quick recruitment of immune cells (including macrophages and neutrophils), and these infiltrating immune cells produce many inflammatory cytokines and growth factors, triggering the activation of myofibroblasts [41]. According to relevant reports, some natural active substances (polysaccharides, protein/peptides, terpenes, sterols, alkaloids, etc.) have functional effects on regulating inflammatory reactions [42]. LF, a naturally occurring nontoxic glycoprotein, induces T-cell activation and inhibits the expression of the proinflammatory factors IL-6 and TNF-α (Figure 2) [43]. These findings suggest that regulating the inflammatory reaction may be a potential mechanism by which LF prevents organ fibrosis.

TGF-β1, a core member of the TGF-β superfamily [44], plays a prominent role in the progression of multiple organ fibrosis. It is considered a critical mediator of organ fibrosis via the activation of its downstream mediators Smad2 and Smad3 [45,46]. Smads can also interact with other inflammation-related signalling pathways, such as the MAPK and NF-κB pathways. These pathways positively or negatively regulate inflammation and organ fibrosis [45]. LF significantly inactivates the MAPK and NF-κB signalling pathways, suppresses the expression of proinflammatory factors (TNF-α and IL-1β), and reduces deoxynivalenol-induced liver inflammation or hyperoxia-induced lung and kidney systemic inflammation [28,47]. In a liver fibrosis rat model stimulated by bile duct ligation, oral LF (300 mg/kg/day) treatment reduced inflammation and fibrosis of the liver via downregulation of the TGF-β1/Smad2 signalling pathway [48]. LF administration also downregulates inflammatory reactions and relieves thioacetamide-induced liver fibrosis by inactivating the TGF-β1 signalling pathway [49]. In summary, LF might be a potential candidate agent for preventing organ fibrosis by modulating the inflammatory process (Figure 2).

### 3.2. Modulating Oxidative Stress

Oxidative stress induced by reactive oxygen species (ROS) has been implicated in the development and progression of many chronic diseases, such as chronic obstructive pulmonary disease, atherosclerosis, cancer, and Alzheimer’s disease [50]. Involved in cell growth, death and differentiation, ROS usually consist of superoxide anions, hyphochlorous acid, hydrogen peroxide, singlet oxygen, hypochlorite, hydroxyl radicals, and lipid peroxides [51]. ROS can bind with and lead to dysfunction of nucleic acids, enzymes, membrane lipids, proteins, and other small molecules [52]. Oxidative stress is involved in the pathogenesis of many diseases by upsetting the balance between free radical production and antioxidant defence [53]. The potential antioxidative effect of LF has been reported [54,55]. LF administration significantly inhibited deoxynivalenol-induced oxidative reactions in the liver [28]. An in vitro study indicated that LF treatment markedly reversed intestinal cell injury upon H_2_O_2_ exposure [56]. The protective effect of LF on mesenchymal stem cells (MSCs) has also been reported. It can reduce oxidative stress and prevent the oxidative stress-induced senescence and apoptosis of MSCs [57]. All of these reports showed that LF has extensive antioxidant activity.

**Figure 3:**
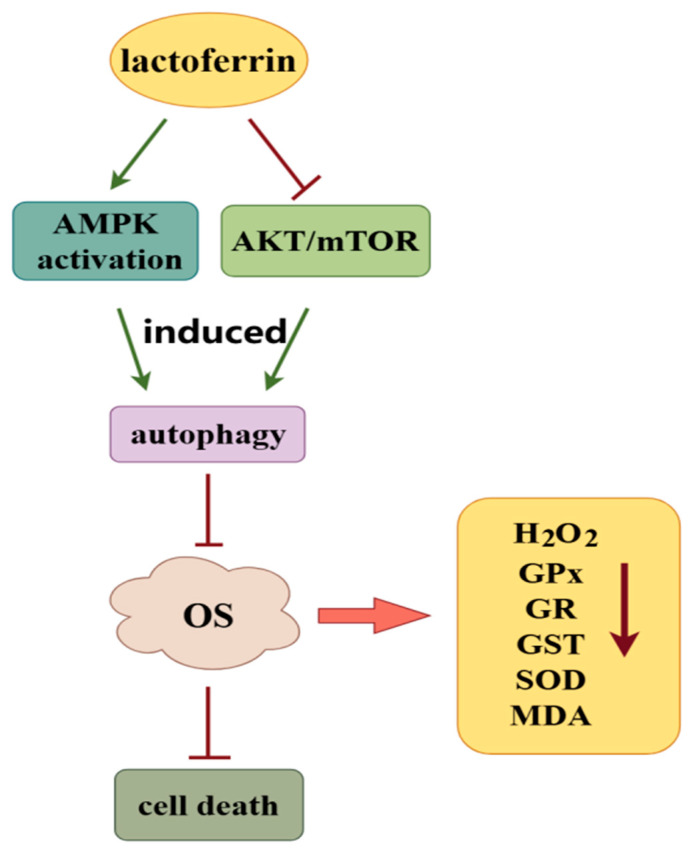
Potential mechanisms by which LF alleviates organ fibrosis by regulating oxidative stress. LF induces autophagy and inhibits oxidative stress-induced cell death and apoptosis by activating AMPK and inhibiting the Akt/mTOR pathway. LF also downregulates the levels of H_2_O_2_, GPx, GR, GST, SOD, and MDA by inhibiting the occurrence of oxidative stress. In the figure, the green arrow represents promotion, the red line represents inhibition, and the deep red arrow represents downregulation.

The antioxidant activity of LF might play an important role in preventing organ fibrosis. LF treatment can induce autophagy by activating AMPK and inactivating the Akt/mTOR signalling pathway, subsequently inhibiting oxidative stress-induced HK-2 cell apoptosis and alleviating renal fibrosis [58]. Thioacetamide exposure led to decreased serum levels of albumin and alkaline phosphatase, as well as decreased liver content of reduced glutathione, triggering liver fibrosis in rats. Oral administration of LF (200 mg/kg/day) to the experimental group of rats significantly reversed thioacetamide-induced reductions in glutathione reduction and malondialdehyde (MDA) accumulation and ultimately alleviated oxidative response-induced liver fibrosis (Figure 3) [59]. 

In a hyperoxia-induced mouse model, aerosolized bovine LF contributed to the suppression of oxidative stress, relieving lung injury and fibrosis [60]. This function was also proven in a multifunctional composite material of LF-decorated cerium oxide nanoparticles. Compared with cerium oxide nanoparticles, LF-decorated cerium oxide nanoparticles possess greater antioxidant activity and alleviate unilateral ureteral obstruction-induced renal fibrosis by inhibiting the TGF-β1 signalling pathway [61]. This finding indicated that oxidative stress was a major reason for the occurrence of organ fibrosis and that LF administration resulted in strong antioxidant activity and significantly relieved organ fibrosis.

### 3.3. Balancing the Synthesis and Degradation of the ECM

**Figure 4:**
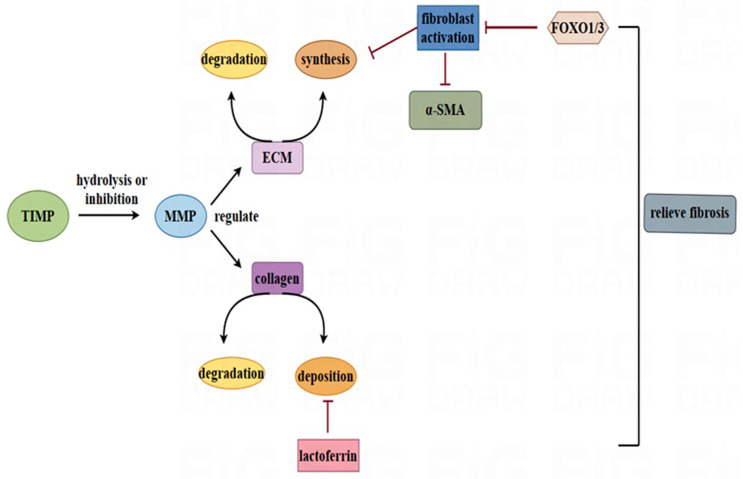
Potential effects of LF on regulating the ECM balance and preventing fibrosis. The balance between ECM synthesis and decomposition is regulated by TIMP and MMP tissue inhibitors. TIMP can also hydrolyse and inhibit MMP production, which can degrade collagen synthesis in various tissues and organs, ultimately preventing fibrosis. In the figure, the black arrow represents promotion, and the red line represents inhibition.

In chronic fibroproliferative diseases, the sensitive balance between the synthesis and degradation of the ECM is disrupted, and continuously activated myofibroblasts produce surplus ECM, causing connective tissue to infiltrate parenchymal tissue [62,63]. Fibrosis is a common age-related disease characterized by excessive formation of fibrous connective tissue. However, prolonged excessive deposition of collagen can destroy normal organic structures and ultimately cause organ failure. In liver tissue, the balance between the synthesis and decomposition of the ECM is regulated by tissue inhibitors of matrix metalloproteinases (TIMPs) and metalloproteinases (MMPs) [64,65]. MMPs are able to directly or indirectly regulate the degradation of the ECM and play significant roles in the formation and regression of liver fibrosis [66]. Studies have shown that MMPs can mediate the degradation of collagen in various tissues and organs, thereby exhibiting the potential to alleviate organ fibrosis. TIMPs have opposite effects and can hydrolyse and inhibit the generation of MMPs. ECM deposition is a pathological characteristic of fibrotic diseases and the central pathological process of fibrosis, indicating that the balance of the MMP–TIMP system and the ECM content has a significant role in preventing organ fibrosis (Figure 4) [65]. Fibroblast proliferation is an important feature, and fundamental changes contribute to ECM synthesis and the formation of excessive fibrous connective tissue [67]; inhibiting fibroblast changes is a possible antifibrotic strategy.

The potential regulatory effects of LF on MMP expression have been studied. As a transactivator of MMP1 transcription, human LF treatment significantly upregulated MMP1 expression in PC-14 cells [68]. TIMP overexpression is a risk factor for organ fibrosis, and the MMP/TIMP ratio is commonly used to assess the risk of organ fibrosis [69]. Clinical trials have shown that administering a compound containing 300 mg LF via the vagina 4 h before amniocentesis can increase MMP-2 activity, reduce TIMP-1 levels, and increase the MMP-2/TIMP-1 molar ratio [70], indicating its potential for promoting ECM degradation and preventing organ fibrosis. However, contrary results in which LF regulates the synthesis and degradation of the ECM have also been reported. In a normal human dermal fibroblast model, bovine LF promoted wound healing by increasing the expression of *COL1A1* mRNA and promoting collagen biosynthesis [71]. During the inflammatory phase, LF treatment also reduces MMP-9 expression in LPS-induced bovine peripheral blood mononuclear cells [72]. The evidence that LF prevents organ fibrosis by balancing the synthesis and degradation of the ECM is still insufficient, and further studies are needed to determine the connection between LF and ECM metabolism during organ fibrosis.

## 4. New Perspectives for Lactoferrin Prevention of Organ Fibrosis

### 4.1. Modulating Microbiota Abundance

Over the last few decades, increasing evidence has suggested that the gut microbiome plays a critical role in regulating both the innate and adaptive immune systems and has a serious impact on the pathogenesis of various immune-mediated diseases [73]. There is a certain relationship between the occurrence and execution of various organ fibrosis diseases and disorders of the gut microbiota. The gut microbiota comprises approximately 100 trillion bacteria from approximately 1000 diverse species. Under normal conditions, these bacteria live in symbiosis with their host and carry out significant and complicated roles in metabolism and immunity [11]. Usually, ecological imbalance is related to whether the integrity of the intestinal barrier is disrupted, which promotes the transfer of bacteria and bacterial products into the circulation, induces systemic activation of immune and inflammatory responses, and leads to direct or indirect damage to tissues [68]. In genetically susceptible hosts, dysregulation of microbiota immune interactions is thought to lead to the development of various immune-mediated diseases [69]. Microbiota dysbiosis is closely related to liver, heart, or lung fibrosis [74,75,76]. Research has indicated that bile duct ligation results in excessive hepatic bile acid and induces liver fibrosis. Treatment with the probiotic *Lactobacillus rhamnosus GG* contributed to reducing inflammation and hepatic bile acid accumulation and preventing bile duct ligation-induced liver fibrosis [30]. In a mouse model of lung fibrosis, Lactobacillus supplementation significantly downregulated collagen 1 A production, inactivated the IL-17A and TGF-β1 signalling pathways, and alleviated bleomycin-stimulated lung fibrosis [77]. In general, a growing body of evidence suggests that an imbalance in the gut microbiota is a main factor in the pathogenesis of disease and is intrinsically connected to the development of organ fibrosis. The microbiota may represent an active promising target for the prevention and treatment of organ fibrosis.

LF, an important iron-containing glycoprotein from milk, strongly modulates the microbiota. A study using an ethanol-induced liver injury mouse model revealed that LF administration increased the abundance of the beneficial bacteria *Akkermansia* and *Lactobacillus*, reduced inflammatory reactions, and alleviated liver injury [78]. Moreover, LF could reverse antibiotic-induced microbiota dysbiosis. In the clindamycin-induced mouse model, the family levels of *Bacteroidaceae*, *Prevotellaceae* and *Rikenellaceae* decreased. However, LF treatment significantly reversed the changes in these bacterial genera at the family level [79]. Studies have also indicated that oral LF supplementation helps maintain the microbiota balance of newborn or paediatric patients with hematologic malignancies receiving chemotherapy [34,80]. In summary, LF can regulate the homeostasis of the gut microbiota, thereby exerting functional effects on various conditions. Importantly, the regulation of the gut microbiota by LF has provided novel research prospects for the prevention of organ fibrosis.

### 4.2. Potential Therapeutic Targets of miRNAs

According to previous studies, miRNAs act as cell regulators in many cell processes, including migration, proliferation, differentiation, and apoptosis [81,82]. Recent research has revealed that miRNAs play important roles in the development of fibrotic diseases [83]. This approach may become a new treatment method for organ fibrosis. miRNAs are a class of small noncoding single-stranded RNAs with lengths of approximately 21–22 nucleotides. They exert a regulatory function via complementary pairing with the 3′-untranslated region (3′-UTR) of the target messenger RNA (mRNA) [84,85]. These findings indicate that miRNAs are potential targets for fibrosis prevention. miR-155 is reported to be an underlying biomarker for liver fibrosis [86]. In a RAW264.7 cell model, LF pretreatment inhibited LPS-induced miR-155 overexpression [87]. LF might regulate miRNA expression and be involved in preventing organ fibrosis; however, more evidence is still needed to prove this potential effect.

## 5. Conclusions

This review highlights the progress in the application of LF for the treatment and prevention of organ fibrosis. Increasing evidence shows that LF has potential antifibrotic effects on many tissues and organs, such as the liver, heart, lung, kidney and skin. This process may involve regulating inflammation, oxidative stress, and the synthesis and degradation of the ECM. However, research into the antifibrotic properties of LFs has focused mostly on animal studies and cell models of fibrosis, and evidence from clinical research is inadequate. Moreover, novel mechanisms by which LF prevents organ fibrosis, such as associations with the microbiome and miRNA regulation, are gradually being studied. As a natural protein, LF may become a prospective therapeutic agent for organ fibrosis in the future.
